# Oxidation of L-Ascorbic Acid in the Presence of the Copper-Binding Compound from Methanotrophic Bacteria *Methylococcus capsulatus* (M)

**DOI:** 10.3390/biomimetics5040048

**Published:** 2020-10-08

**Authors:** Lidia V. Avdeeva, Rudolf I. Gvozdev

**Affiliations:** Institute of Problems of Chemical Physics of RAS, Academician Semenov Ave. 1, Chernogolovka 142432, Moscow Region, Russia; rigvozdev@gmail.com

**Keywords:** antioxidant, chalkophores, methanotrophs, L-ascorbic acid, mimetic oxidation

## Abstract

The oxidation of ascorbic acid by air oxygen and hydrogen peroxide in the presence of the copper-binding compound (cbc) from bacteria *Methylococcus capsulatus* (M) was studied. The rate constant of ascorbic acid oxidation by air oxygen in the presence of the copper complex with cbc from *M. capsulatus* (M) was shown to be 1.5 times higher than that of the noncatalytic reaction. The rate constant of ascorbic acid oxidation by hydrogen peroxide in the presence of the copper complex with cbc from *M. capsulatus* (M) decreased by almost one-third compared to the reaction in the absence of the copper complex with cbc. It was assumed that cbc can be involved in a multilevel system of antioxidant protection and can protect a bacterial cell from oxidation stress. Thus, the cbc is mimetic ascorbate oxidase in the oxidation of ascorbic acid by molecular oxygen.

## 1. Introduction

Methanotrophic bacteria (methanotrophs) use methane as a source of carbon and energy and participate in the global carbon cycle. Biological metabolism of methane starts from the activation of the inert C–H bond of methane catalyzed by methane monooxygenase (MMO). There are two types of methane monooxygenase: soluble (sMMO) and particulate (pMMO). An interrelation is observed between the copper concentration and the expression of two monooxygenases. Low-molecular-weight copper-binding chromopeptide named methanobactin (mb) is synthesized by methanotrophs to absorb copper [1]. Methanotrophs are widely abundant in nature and live under various conditions. Changes in external factors lead to a series of consequences for a bacterial cell, in particular, in an increase in the level of reactive oxygen species (ROS) in the cell, among which are superoxide (O_2_^−^), singlet oxygen, hydrogen peroxide (H_2_O_2_), hydroxyl radical (OH^−^), and others. Hydrogen peroxide is the most stable of the intermediate products of oxygen reduction but is the least reactive one. It is known that hydrogen peroxide is a reversible inhibitor for pMMO from *Methylosinus trichosporium* OB3b under anaerobic conditions [2].

Methanotrophs have several sources of ROS. Of course, ROS are constantly formed in a living cells as a result of various reactions, but they are also formed as products of normal oxygen metabolism. The enhancement of ROS formation in methanotrophs can be a consequence of an increase in the intensity of metabolic processes or rising of cultivation temperature. For example, *Methylocaldum szegediense* O-12 and *Methylococcus capsulatus* Bath form ROS at optimum temperatures more intensively than at suboptimum temperatures [3]. Hydrogen peroxide and other ROS are formed due to methane oxidation catalyzed by MMO and as a result of the oxygenase activity of ribulose bisphosphate carboxylase (RubisCO) [2,4]. It is shown that pMMO from *M. trichosporium* OB3b forms hydrogen peroxide in the presence of duroquinol under anaerobic conditions [2].

The specialized antioxidant systems, whose function is reduced to the inactivation of free radicals, maintain the ROS levels in physiological limits. Methanotrophs *M. szegediense* O-12 and *M. capsulatus* Bath contain glutathione peroxidase, superoxide dismutase, and cytochrome *c* peroxidase as the antioxidant system [3]. The pigment melanin in methanotroph *M. szegediense* O-12 characterized by peroxide decomposing activity also has antioxidant properties [5]. It is shown that mb and Cu-mb from *M. trichosporium* OB3b reduce oxygen to superoxide in the presence of a reductant [6]. The dismutation of superoxide anion O_2_˙^−^ to H_2_O_2_ and reduction of H_2_O_2_ to H_2_O without hydroxyl radical (OH˙) formation can be induced by mb [6]. It is known that mb from *Methylocystis* strain SB2 possess similar properties [1]. This is assumed to be a common property of all mb [1]. However, not all methanotrophs can synthesize mb [7,8]. The existence of genes that encode mb only in several strains of methanotrophs referring to phylum *Alphaproteobacteria* has been proved [7]. The strains referred to phylum *Gammaproteobacteria* synthesize another class of chalkophores [9,10]: copper-binding compounds (cbc). The cbc was initially identified in the methanotroph *M. capsulatus* Bath in association with pMMO [11]. In cells cultured in copper-free medium, the cbc was predominately observed in the extracellular fraction. The cbc isolated from the spent media did not contain copper. Copper-containing cbc was originally proposed as a cofactor of the pMMO, based on the irreversible loss of pMMO activity following separation [11]. The cbc from *M. capsulatus* Bath and *M. album* BG8 differed from the mb from *M. trichosporium* OB3b in molecular mass and spectral properties. The mb from *M. trichosporium* OB3b can remove Cu(I) from the Cu-cbc from *M. capsulatus* Bath and *M. album* BG8 [10]. The EPR and kinetic experiments suggest that Cu–cbc is a redox-active chromopeptide that stimulates methane oxidation by pMMO [12]. The explanation and understanding biological properties of the cbc is important task. Therefore, the purpose of this work is to study of the redox active properties of the cbc from *Methylococcus capsulatus* (M), in particular in the oxidation reaction of L-ascorbic acid (H_2_A) in order to highlight the properties of the cbc for understanding its function in methane oxidation by pMMO and possible participation in the antioxidant system of methanotrophs.

## 2. Materials and Methods

### 2.1. Cells Growth

Cells of *M. capsulatus* (M) were grown in the flow cultivation regime in an Ankum 2M fermenter (Institute for Biological Instrumentation of the Russian Academy of Sciences, Pushchino, Russia) on a standard mineral medium containing CuSO_4_ (5 × 10^−6^ mol L^−1^) at 42 °C and pH 5.6 as described previously [13]. The cells were sedimented by centrifugation at 6000 g for 30 min on an OPn-8 centrifuge (Dastan, Bishkek, Kyrgyzstan).

### 2.2. Copper-Binding Compound Isolation

The isolation of the cbc was conducted from the cultural liquid of *M. capsulatus* (M) using liquid chromatography on a column packed with Diaion HP20 (Supelco) as previously described [6]. Elution was carried out with 60% MeOH: 40% H_2_O. The obtained preparation of cbc was lyophilized [14]. The cbc complex with copper (Cu-cbc) was synthesized by the incubation of cbc with an aqueous solution of copper sulphate [9]. Copper titrations were determined by addition of 1 × 10^−3^ mol L^−1^ or 10 × 10^−3^ mol L^−1^ aqueous stock solutions of CuSO_4_ to 5 × 10^−3^ mol L^−1^ cbc dissolved in 0.05 M sodium acetate buffer (pH 5.5). All titrations were determined at room temperature under aerobic conditions. All glassware was soaked in 1 N HNO_3_ for 15 h then rinsed with copious amounts of distilled-deionized water. Copper was added to solutions of cbc, mixed and incubated for 5 min before spectral determinations [9]. The formation of the complex was monitored by fluorescence quenching method every 45 s for 5–15 min (Figure S1) using a Cary-Eclipse spectrofluorimeter (Varian, Mulgrave, Australia) [15]. Excitation wavelengths 280, 330, and 400 nm were used. Between scans, the samples were stored in the dark to avoid photodegradation.

### 2.3. Oxidation of L-Ascorbic Acid

The oxidation of L-ascorbic acid (7 × 10^−5^ mol L^−1^) by hydrogen peroxide (0.64 × 10^−3^ mol L^−1^) and air oxygen was conducted in a medium of 0.05 M sodium acetate buffer (pH 5.5, a volume of 3 mL) at room temperature (approximately 21 °C). The reactions in the presence of Cu-cbc (7 × 10^−6^ mol L^−1^) or an aqueous solution of copper sulphate (6 × 10^−6^ mol L^−1^) were carried out in parallel experiments. Solutions of hydrogen peroxide were prepared by the dilution of a 30% solution of H_2_O_2_. The concentration of hydrogen peroxide was determined by spectrophotometric titration of the reaction of hydrogen peroxide with potassium permanganate in concentrated sulphuric acid [16,17]. The solutions of all reagents were freshly prepared in degassed distilled-deionized water and kept in darkness. The oxidation of ascorbic acid was detected by a decrease in the absorbance at 265 nm (ε = 14,192 ± 507 L mol^−1^ cm^−1^) [17] using a Specord M40 spectrophotometer (Carl Zeis Industrielle Messtechnik GmbH, Oberkochen, Germany) equipped with the software (Institute of Problems of Chemical Physics of RAS, Chernogolovka, Russia) (Figure S2). The reaction was carried out in a quartz cell of the spectrophotometer. The reaction was initiated by ascorbic acid. Time of observation of spectral changes for calculation of kinetic parameters was *t*_1_/_2_. The literature data show that, irrespective of the nature of the oxidizer (O_2_ or H_2_O_2_), which concentration are constant the oxidation of H_2_A satisfy the pseudo-first-order equation [17]. The observed rate constants (*k_obs_*) of H_2_A oxidation were calculated by Equation (1) of the pseudo-first order [17]

(1) $$k_{obs}=\frac{1}{t}\ln\left[\frac{c^{0}}{c^{t}}\right]=\frac{1}{t}\ln\left[\frac{A_{0}}{A_{t}}\right],$$

where *A*_0_, *A_t_* and *c*^0^, *c^t^* are the absorbances and concentrations, respectively, at the initial moment and moment *t*.

The half reaction time (*t*_1/2_) was calculated by Equation (2).

(2) t12=1kobsln[c012c0],

The reaction rate of decrease in H_2_A (*w*_H2A_) was calculated by Equation (3).

(3) $$w_{H_{2}A}=-\frac{d[H_{2}A]}{dt},$$

## 3. Results

**Figure 1:**
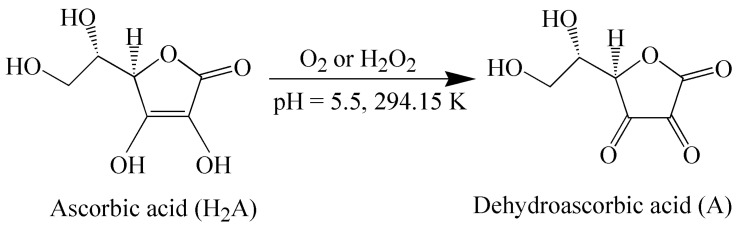
Scheme of ascorbic acid oxidation.

The oxidation reactions of ascorbic acid (H_2_A) by air oxygen and hydrogen peroxide were studied to elucidate the oxidase properties and to reveal the role of cbc in the oxidation of organic compounds. Ascorbic acid (C_6_H_8_O_6_) is an organic acid with the antioxidant properties acting in chemical and biological systems. Only the L-enantiomer exhibits biological activity. The general scheme of the reaction is presented in Figure 1.

**Figure 2:**
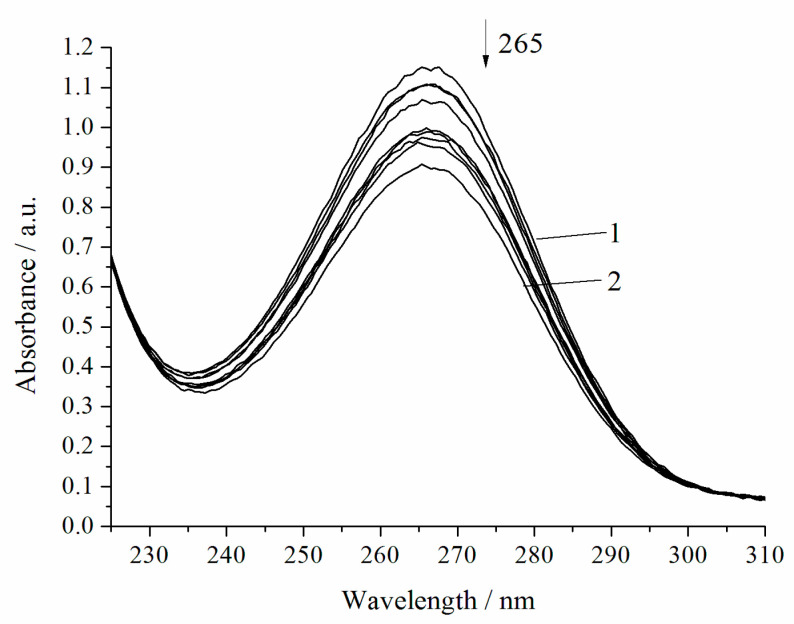
Spectrophotometric changes registered for the oxidation of H_2_A (*c*^0^ = 7 × 10^−5^ mol L^−1^) by air at 294.15 K and pH 5.5; t, s: 1, 0; 2, 1200. Arrow shows the direction of the spectral evolution.

The oxidation of H_2_A to dehydroascorbic acid (A) occurs due to the donation of two protons and two electrons. The reaction of H_2_A oxidation assumes the direct interaction of the ascorbate monoanion (HA^−^) with the oxygen. An aqueous solution of H_2_A is stable in the absence of oxygen, whereas, in air, the solution is stable at pH 5–6 [18]. The oxidation of H_2_A was carried out in a medium of 0.05 M sodium acetate buffer (pH 5.5) in order to minimize the noncatalytic decomposition of H_2_A. The initial solution of H_2_A (1 × 10^−2^ mol L^−1^) used in the work is relatively stable [19]. The study was carried out at a H_2_A concentration lower by three orders of magnitude (7 × 10^−5^ mol L^−1^). This solution of H_2_A is already less stable, and the noncatalytic oxidation of H_2_A by air oxygen with an appreciable rate is observed (Figure 2, Table 1).

The mechanism of the noncatalytic oxidation of H_2_A by molecular oxygen in an aqueous solution and in methanol is already known [20]. At a neutral pH, the reaction starts from the interaction of the ascorbate monoanion (HA^−^) with an oxygen molecule to form the oxygen adduct of H_2_A. Then, the reaction can proceed via two routes, one of which (most probable) is the disproportionation and formation of superoxide radical (O_2_^−^) and monodehydroascorbic acid radical (HA^−^). The different mechanism via dehydroascorbic acid radical (A^−^) formation was proposed earlier for the spontaneous oxidation of H_2_A [21]. Both mechanisms proposed for H_2_A oxidation assume the direct interaction of the ascorbate monoanion with the oxygen molecule.

**Figure 3:**
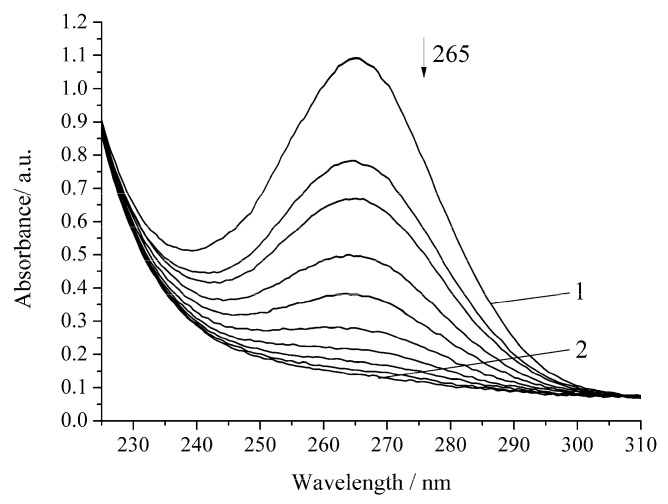
Spectrophotometric changes registered for the oxidation of a solution of H_2_A (*c*^0^ = 7 × 10^−5^ mol L^−1^) by H_2_O_2_ (*c*^0^ = 0.64 × 10^−3^ mol L^−1^) at 294.15 K and pH 5.5; t, s: 1, 0; 2, 1200. Arrow shows the direction of the spectral evolution.

The noncatalytic oxidation of H_2_A occurs rapidly in the presence of hydrogen peroxide (Figure 3, Table 1). Under the chosen conditions, the complete decomposition of H_2_A occurs within 20 min. Regardless of the nature of the oxidant (oxygen or hydrogen peroxide), the reactions of H_2_A oxidation obey the pseudo-first-order equation [17].

It is known that, on the one hand, H_2_A is a strong reductant (*E*^0^_A/H2A_ = 0.390 V vs. N.H.E.) and, on the other hand, a weak acid dissociating via two steps, as follows [18]:

H_2_A ↔ HA^−^ + H^+^, p*K*_1_ = 4.04

HA^−^ ↔ A^2−^ + H^+^, p*K*_2_ = 11.34

where H_2_A is ascorbic acid, HA^−^ is ascorbate monoanion, and A^2−^ is dehydroascorbic acid.

Therefore, at pH 5.5, H_2_A almost completely exists in the solution as an ascorbate monoanion (HA^−^). All three forms of H_2_A are active, and the activity order is the following: H_2_A < HA^−^ < A^2−^ [22]. The ascorbate monoanion (HA^−^) is fairly rapidly dissociated to dehydroascorbic acid (A^2−^), which determines the instability of H_2_A (Figure 2).

**Figure 4:**
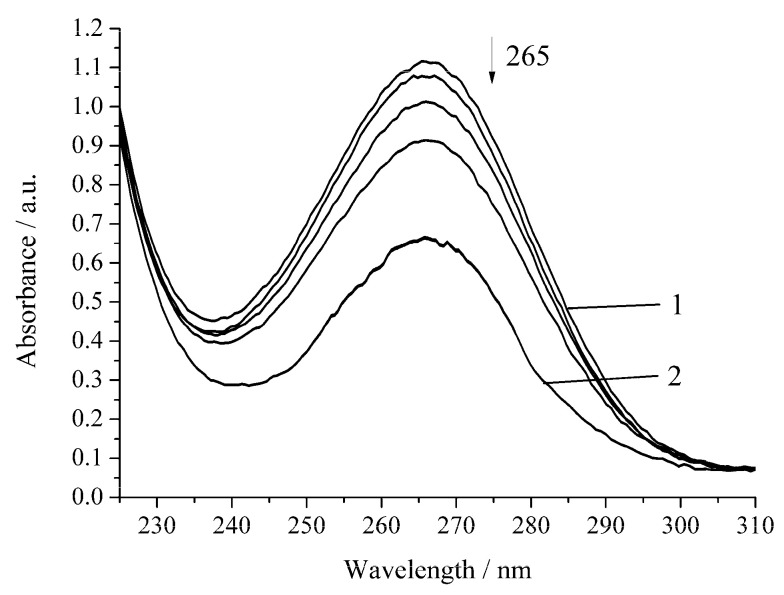
Spectrophotometric changes registered for the oxidation of a solution of H_2_A (*c*^0^ = 7 × 10^−5^ mol L^−1^) by air oxygen in the presence of Cu-cbc (7 × 10^−6^ mol L^−1^) from *M. capsulatus* (M) at 294.15 K and pH 5.5; t, s: 1, 0; 2, 1620. Arrow shows the direction of the spectral evolution.

In the presence of Cu-cbc from *M. capsulatus* (M), the oxidation rate of H_2_A by air oxygen increases by 1.5 times compared to the noncatalytic oxidation of H_2_A (Figure 4, Table 1).

**Figure 5:**
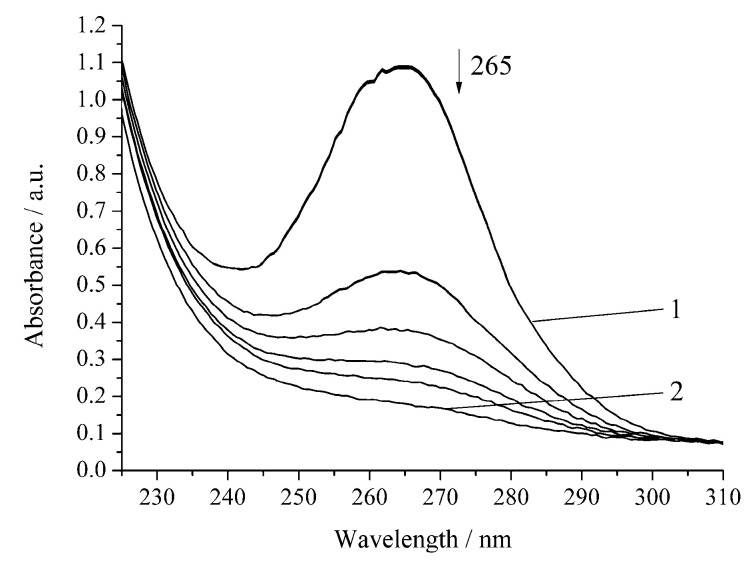
Spectrophotometric changes registered for the oxidation of a solution of H_2_A (*c*^0^ = 7 × 10^−5^ mol L^−1^) by H_2_O_2_ (*c*^0^ = 0.64 × 10^−3^ mol L^−1^) in the presence of Cu-cbc (7 × 10^−6^ mol L^−1^) from *M. capsulatus* (M) at 294.15 K and pH 5.5; t, s: 1, 0; 2, 1140. Arrow shows the direction of the spectral evolution.

However, the inhibition of the reaction is observed for the oxidation of H_2_A by hydrogen peroxide in the presence of Cu-cbc (Figure 5, Table 1).

**Figure 6:**
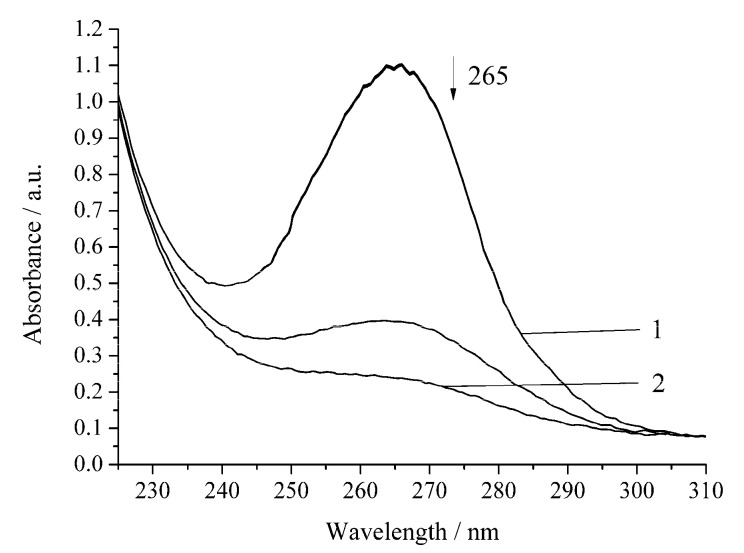
Spectrophotometric changes registered for the oxidation of a solution of H_2_A (*c*^0^= 7 × 10^−5^ mol L^−1^) by air oxygen in the presence of Cu(II) (6 × 10^−6^ mol L^−1^) at 294.15 K and pH 5.5; t, s: 1, 0; 2, 240. Arrow shows the direction of the spectral evolution.

**Figure 7:**
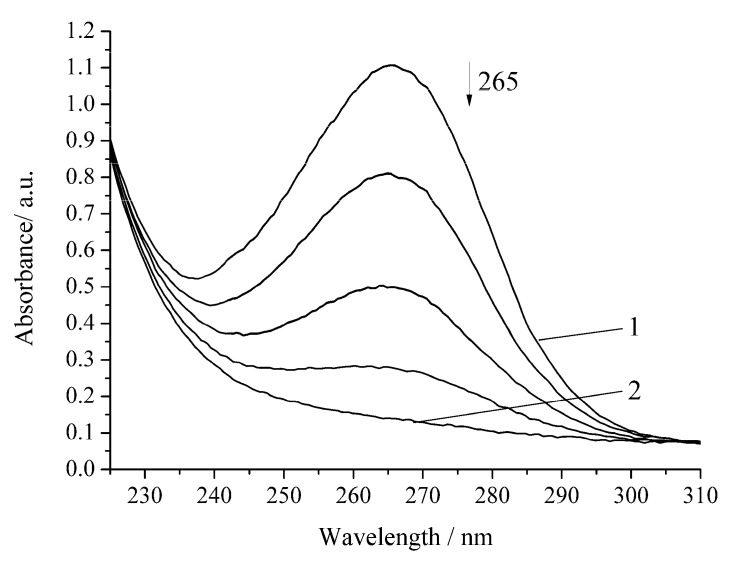
Spectrophotometric changes registered for the oxidation of a solution of H_2_A (*c*^0^ = 7 × 10^−5^ mol L^−1^) by H_2_O_2_ (*c*^0^ = 0.64 × 10^−3^ mol L^−1^) in the presence of Cu(II) (6 × 10^−6^ mol L^−1^) at 294.15 K and pH 5.5; t, s: 1, 0; 2, 180. Arrow shows the direction of the spectral evolution.

The oxidation rate of H_2_A by hydrogen peroxide in the presence of Cu-cbc is higher than that for oxidation by air oxygen as in the case of the noncatalytic oxidation of H_2_A. However, the rate constant is lower by 37% than that of the similar reaction without Cu-cbc (Table 1). This reaction was carried out in the presence of the copper complex with cbc obtained by the titration of a solution of cbc with a solution of copper sulphate. It is known that transition metals (Cu(II), Fe(II), V(V), Cr(VI), and Mn(II)) accelerate the oxidation of H_2_A [23]. Copper has the highest activity among the studied metals [21,23]. It is known that copper also catalyzes the oxidation of H_2_A in an acidic medium by peroxomonosulphate [24] and peroxodiphosphate [25]. The oxidation of H_2_A by copper(II) in an alkaline solution was studied by the chemiluminescence method using a luminol/copper(II) system. Oxygen and halide anions (Cl^−^, Br^−^, and I^−^) significantly catalyze this oxidation of H_2_A to form ROS (H_2_O_2_ and O_2_^−^) [26]. In order to exclude that, the observed effect (Figure 4 and Figure 5) can be induced by copper cations (added for formation of the Cu-cbc complex); the oxidation of H_2_A was conducted in the presence of copper(II) sulphate under the same conditions (Figure 6 and Figure 7).

In the presence of copper(II), high rates for H_2_A oxidation are observed in the cases of both air oxygen and hydrogen peroxide (Table 1). It is known that copper(II) forms complex(es) with H_2_A, which was confirmed kinetically [25]. The mechanism was proposed for the catalytic oxidation of H_2_A by copper cations [21]. The reaction mechanism of the catalytic oxidation of H_2_A is accompanied by the formation of radical species, as is the case for noncatalytic oxidation.

The numerical values of the kinetic parameters for H_2_A oxidation obtained in this paper are presented in Table 1.

The reaction of full H_2_A oxidation in all the studied systems takes place during different times (Figure 2, Figure 3, Figure 4, Figure 5, Figure 6 and Figure 7) and have different reaction rates (*w*_H2A_) of H_2_A oxidation and different half-rotation times (*t*_1/2_), which do not depend on the initial concentration of the H_2_A (Table 1). The time of half-rotation of the H_2_A by air oxygen without Cu-cbc is maximum and decreases by 35% in the presence of Cu-cbc. The data in Table 1 show that the oxidation of H_2_A by hydrogen peroxide is characterized by the rate constant exceeding that of the analogous oxygen oxidation by approximately ten times. This can be explained by a high activity of peroxide radicals [27]. Among all reactions studied in this work, the highest catalytic effect was observed with copper cations for the oxidation by both oxygen and hydrogen peroxide (Table 1). The “theoretical” concentration of free copper if there was no binding of copper with cbc in the case of the reactions with Cu-cbc (the copper concentration calculated from the amount of copper consumed to the titration of a solution of cbc, taking into account all dilutions) should be 7 × 10^−6^ mol L^−1^, which is comparable with the copper concentration used in the studied reactions (6 × 10^−6^ mol L^−1^) (Figure 6 and Figure 7). However, the catalytic effect of copper cations is much higher than that of Cu-cbc (Table 1). The activity of copper(II) in the oxidation of H_2_A can be caused by three factors. First, copper(II) cations in a solution are more accessible than Cu-cb*c* for the formation of intermediate compounds with the reagents [19]. Second, copper(II) cations in this concentration are not active in the decomposition of hydrogen peroxide and almost do not change the concentration of the latter [19]. Third, differences are possible in the mechanisms of H_2_A oxidation by copper cations and Cu-cbc.

The mechanism and kinetics of H_2_A oxidation by different oxidizing agents and catalysts are the subjects of extensive studies [19,20,21,22,23,24,25,26,28]. A considerable contribution of radical reactions to the oxidation of H_2_A is presently doubtless. However, no unambiguous mechanism was proposed for either hydrogen peroxide decomposition or H_2_A oxidation. This is due to the fact that many factors affect these processes: pH of the solution, temperature, impurities. The oxidation of H_2_A is substantially accelerated in the presence of cations and complexes of Co(II), Fe(II), Cu(II), Mn(II), and Ni(II) [19,21,22,23,24,25,26,28]. Other copper complexes exhibiting the catalytic properties in reaction oxidation of organic compounds are also known. The activity of the complexes [Cu(L^1^/L^2^)]-[MCl_4_] in the oxidation of H_2_A by molecular oxygen in an aqueous solution was studied [17]. It is also known that copper phthalocyanine (CuPcCl_15_) catalyzes cyclohexane oxidation by hydrogen peroxide to cyclohexanol and cyclohexanone [29]. The copper(II) salts (acetate, perchlorate, and chloride) catalyze the oxidation of benzene by hydrogen peroxide to phenol and quinine [30]. However, Cu-cbc give the inverse effect, and inhibition of reaction H_2_A oxidation by hydrogen peroxide was observed (Table 1).

Now, it is difficult to propose a mechanism of the catalytic effect of Cu-cbc on the oxidation of H_2_A. Probably, Cu-cbc catalyzes H_2_A oxidation by oxygen via a mechanism different from the mechanism of catalyzed by the copper(II) cations and its complexes. This is indicated by the inhibition of a similar reaction of H_2_A oxidation by hydrogen peroxide, because this reaction is catalyzed by copper(II) cations. It can be assumed that Cu-cbc, as well as mb, is a redox-active molecule. The Cu-dependent oxidase activity of mb take place in the reduction of O_2_ to O_2_^−^ with either NADH or duroquinol as a reductant. The chemical or biological dismutation of superoxide to hydrogen peroxide was proposed [6]. The hydrogen peroxide reductase activity of mb was shown. In the presence of a reductant, H_2_O_2_ was reduced by Cu-mb. Similar properties are also characteristic of cbc from both *M. capsulatus* Bath and *M.* *album* BG8 [6]. Assuming that Cu-cbc from *M. capsulatus* (M) possesses resembling properties and can form superoxide radical and hydrogen peroxide, this can form a basis of the mechanism of the catalytic activity of Cu-cbc in the oxidation of H_2_A by oxygen.

The oxidation rate (*w*_H2A_) and half-rotation time (*t*_1/2_) of H_2_A oxidation by hydrogen peroxide in the presence of Cu-cbc is lower compared to a similar reaction in the absence of Cu-cbc (Table 1). In addition, deviations from the kinetic curve of H_2_A oxidation from the law of the first order are observed. At small times of transformation of H_2_A, *k_obs_* was 20% more than at large times. These deviations can be satisfactorily described by taking the first-order decomposition of hydrogen peroxide with an effective velocity constant of the order of 50 × 10^−4^ s^−1^. The initial value of *k_obs_* = 12.4 × 10^−4^ s^−1^ is 30% less than its value when H_2_A is oxidized with hydrogen peroxide in a non-metal system. This unexpected result means that the Cu-cbc complex can somehow effectively bind intermediate active particles under the conditions of H_2_A by hydrogen peroxide oxidation and use them primarily for the catalytic reaction of hydrogen peroxide decomposition. Note that copper (II) cations in the solution at the concentration used are not active in the decomposition of hydrogen peroxide and practically do not change its concentration. Thus, we can make an assumption about the high affinity of the Cu-cbc complex to ROS. This may be the reason for the increased rate of methane hydroxylation by pMMO in the presence of Cu-cbc due to the availability of active oxygen-containing copper complexes for a small molecule CH_4_ [12].

The biosynthesis of cbc from *M. capsulatus* (M) depends on the level of copper in the growth medium. An increase in the secretion of cbc to the growth medium is observed at an increased copper content in the growth medium [31]. As iron, copper in oxygen-containing solutions can generate various toxic ROS via the Fenton and Haber–Weiss reactions [1,28]. The formation of ROS is also observed due to the active metabolism of methanotrophs [3]. The ROS levels in cells are maintained in physiological limits by the functioning of specialized protection systems of catalase, superoxide dismutase, cytochrome *c* peroxidase, glutathione peroxidase, and free glutathione [3]. Genes that encode catalase are absent from the genome relative to *M. capsulatus* (M) and well-studied methanotroph *M. capsulatus* Bath [3,32]. No catalase activity was observed for *M. szegediense* O-12 [3]. Genome *M. capsulatus* Bath contains the gene that encodes glutathione peroxidise [32]. Bacterial cytochrome *c* peroxidase in *M. capsulatus* Bath involved in the detoxication of hydrogen peroxide by reducing the latter to water was characterized [3,33]. The obtained results suggest that the cbc not only binds copper, thus diminishing its toxicity, but also the Cu-cbc complex can participate, along with the enzymatic antioxidant systems of methanotrophs, in ROS detoxication caused by an increased copper content in the growth medium and the metabolism of methanotrophs. The mechanism of the action of Cu-cbc in the oxidation of H_2_A will be elucidated elsewhere.

## 4. Conclusions

The biological and physiological properties and functions of mb, different chalkophores and copper-binding compounds (cbc) are probably wider than it seems at first glance. It was shown, for the first time, that the copper complex with the cbc from *M. capsulatus* (M), depending on the nature of the oxidant (oxygen or hydrogen peroxide), can show different properties in reactions of H_2_A oxidation. Namely, the rate constant of H_2_A oxidation in the presence of the copper complex of cbc from *M. capsulatus* (M) by air oxygen was shown to be 1.5 times higher, but it is decreased by almost one-third in oxidation by hydrogen peroxide compared than that of the noncatalytic reactions. This indirectly indicates that the cbc can participate in the detoxication of the reduction products of molecular oxygen, in particular, the formed hydrogen peroxide, which is reduced to water. Thus, the *cbc* is mimetic ascorbate oxidase in the oxidation of H_2_A by molecular oxygen.

## Figures and Tables

**Table 1:** Kinetic parameters for the oxidation of H_2_A (0.05 M sodium acetate buffer, pH 5.5, 294.15 K).

System	*k_obs_* × 10^−4^ (s^−1^)	*t*_1/2_ (s)	*w*_H2A_ × 10^−8^ (mol L^−1^ s^−1^)
H_2_A + Air	1.99 ± 0.14	3497.49	1.34 ± 0.09
H_2_A + Air + Cu-cbc	3.03 ± 0.15	2287.13	1.72 ± 0.08
H_2_A + Air +Cu(II)	48.93 ± 4.91	141.63	14.41 ± 1.45
H_2_A + H_2_O_2_	18.47 ± 1.29	375.20	4.75 ± 0.12
H_2_A + H_2_O_2_ + Cu-cbc	11.57 ± 0.57	598.96	3.96 ± 0.19
H_2_A + H_2_O_2_ + Cu(II)	98.35 ± 9.87	70.46	14.90 ± 1.51

Results are presented as mean values of three replicates ± SD.

## Supplementary Materials

The following are available online at https://www.mdpi.com/2313-7673/5/4/48/s1, Figure S1: Change in fluorescence spectra during titration of stock solutions of cbc from *M. capsulatus* (M) with a solution of copper sulfate; Figure S2: Absorption changes at 265 nm registered for the oxidation of a solution of H_2_A (*c*^0^ = 7 × 10^−5^ mol L^−1^).
