# Supplementary material for: Oxidation of L-Ascorbic Acid in the Presence of the Copper-Binding Compound from Methanotrophic Bacteria Methylococcus capsulatus (M)
Source: Biomimetics (Basel). 2020 Oct 8;5(4):48. doi: 10.3390/biomimetics5040048 (PMC7709488; doi:10.3390/biomimetics5040048)
Supplement: Supplementary file 1 [file biomimetics-05-00048-s001.pdf]

Oxidation of L-ascorbic Acid in the Presence of the Copper-binding Compound from Methanotrophic Bacteria *Methylococcus capsulatus* (M)

Lidia V. Avdeeva * and Rudolf I. Gvozdev

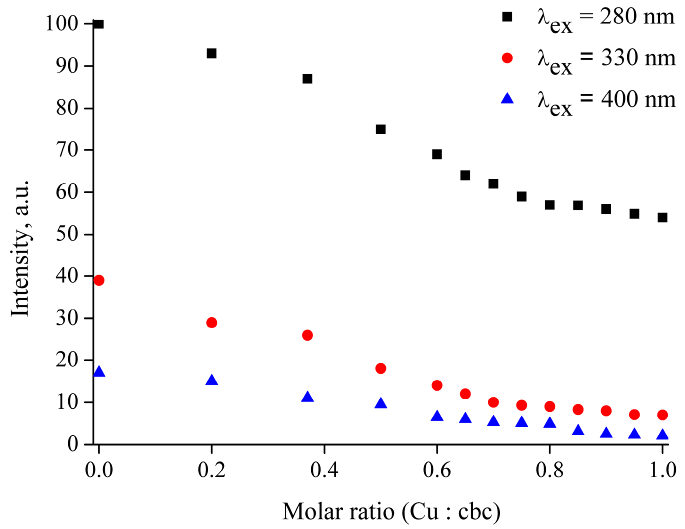

Figure S1. Change in fluorescence spectra during titration of stock solutions of cbc from *M. capsulatus* (M) with a solution of copper sulfate.

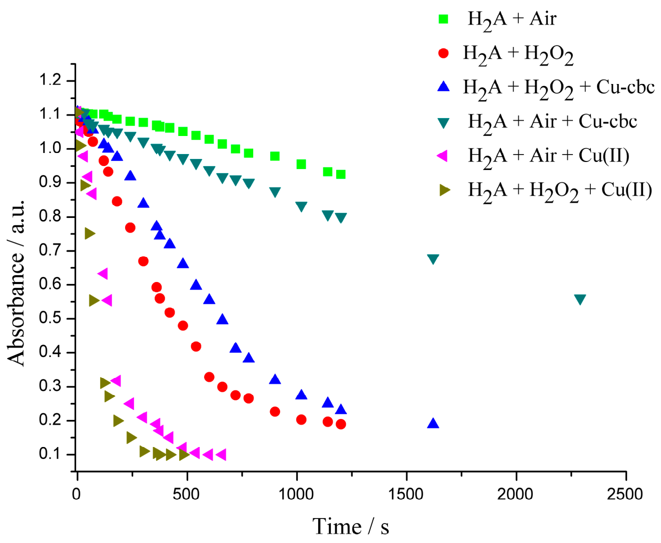

Figure S2. Absorption changes at 265 nm registered for the oxidation of a solution of H_2A ($c^0 = 7 \times 10^{-5}$ mol L^{-1}).
